# The role of microRNAs in pregnancies complicated by maternal diabetes

**DOI:** 10.1042/CS20230681

**Published:** 2024-09-18

**Authors:** Manon D. Owen, Margeurite G. Kennedy, Rachel C. Quilang, Eleanor M. Scott, Karen Forbes

**Affiliations:** 1 Discovery and Translational Science Department, Leeds Institute of Cardiovascular and Metabolic Medicine, Faculty of Medicine and Health, University of Leeds, Leeds, U.K.; 2 Anthony Nolan Research Institute, Royal Free Hospital, Hampstead, London, U.K.; 3 UCL Cancer Institute, Royal Free Campus, London, U.K.; 4 Department of Immunology, Leiden University Medical Center, Leiden, Netherlands; 5 Division of Clinical and Population Sciences, Leeds Institute of Cardiovascular and Metabolic Medicine, Faculty of Medicine and Health, University of Leeds, Leeds, U.K.

**Keywords:** diabetes, miRNA, Placenta

## Abstract

With the global prevalence of diabetes increasing, more people of reproductive age are experiencing hyperglycaemic pregnancies. Maternal Type 1 (T1DM) or Type 2 (T2DM) diabetes mellitus, and gestational diabetes mellitus (GDM) are associated with maternal cardiovascular and metabolic complications. Pregnancies complicated by maternal diabetes also increase the risk of short- and long-term health complications for the offspring, including altered fetal growth and the onset of T2DM and cardiometabolic diseases throughout life. Despite advanced methods for improving maternal glucose control, the prevalence of adverse maternal and offspring outcomes associated with maternal diabetes remains high. The placenta is a key organ at the maternal–fetal interface that regulates fetal growth and development. In pregnancies complicated by maternal diabetes, altered placental development and function has been linked to adverse outcomes in both mother and fetus. Emerging evidence suggests that microRNAs (miRNAs) are key molecules involved in mediating these changes. In this review, we describe the role of miRNAs in normal pregnancy and discuss how miRNA dysregulation in the placenta and maternal circulation is associated with suboptimal placental development and pregnancy outcomes in individuals with maternal diabetes. We also discuss evidence demonstrating that miRNA dysregulation may affect the long-term health of mothers and their offspring. As such, miRNAs are potential candidates as biomarkers and therapeutic targets in diabetic pregnancies at risk of adverse outcomes.

## Introduction

With maternal diabetes currently affecting 21.1 million live births worldwide and the global prevalence of diabetes estimated to surge in the next 20 years [1], the influence of diabetes and associated hyperglycaemia on maternal health and the developing fetus is of increasing concern. Of the pregnancies affected by maternal hyperglycaemia, 19.7% are attributed to pre-existing maternal diabetes (PGDM), including Type 1 diabetes mellitus (T1DM) or Type 2 diabetes mellitus (T2DM), with gestational diabetes mellitus (GDM) being responsible for the remaining 80.3% cases [1]. Pregnancies complicated by PGDM and GDM are associated with short- and long-term adverse outcomes for both mother and offspring. Indeed, maternal hyperglycaemia accelerates the development of various comorbidities associated with diabetes, including diabetic retinopathy and cardiovascular disease [2,3]. These comorbidities are more common in individuals with PGDM compared with GDM, although they can occur in both [4,5]. Moreover, it is known that a diagnosis of GDM during pregnancy increases the risk of post-partum maternal T2DM onset by 7-fold [6]. People with PGDM or GDM have also been identified as a high-risk group for the development of preeclampsia [7,8] a disorder that poses risks to the growing fetus through its constraints on the oxygen supply to the placenta, as well as causing systemic inflammatory stress to maternal organs [9]. Preeclampsia is also associated with an increased maternal risk of developing cardiovascular diseases such as heart disease, stroke and hypertension post-partum [9].

Maternal diabetes not only adversely affects maternal health but is also associated with altered fetal growth, where neonates are more likely to be classed as large-for-gestational-age (LGA) (weighing above the 90th percentile of what is expected for their gestational age) [10–13]. At the opposite end of the spectrum, pregnancies complicated by maternal diabetes are also more likely to produce newborns that are small-for-gestational-age (SGA) (weighing below the 10th percentile of what is expected for their gestational age) compared with healthy pregnancies and can occur as a consequence of fetal growth restriction [14–16]. The aberrant fetal development observed in pregnancies affected by PGDM and GDM can also manifest as congenital abnormalities unrelated to birthweight, for example, cardiovascular and neural tube developmental defects are common in newborns exposed to hyperglycaemia *in utero* [2,7,17]. Birth defects are established early in fetal development, during organogenesis, and therefore the timing of the onset of maternal hyperglycaemia has a major impact on organogenesis and the risk of congenital anomalies [18]. This is illustrated by the markedly increased risk of congenital malformations witnessed in cases of PGDM, and the moderately increased risk seen in cases of GDM, as compared with normal pregnancies [19]. These risk differences reflect the clinical distinction of PGDM and GDM, where PGDM manifests as poor glycaemic control prior to conception, compared with GDM, where glycaemic control deteriorates during gestation. Although the exact mechanism is unknown, it is suspected that the hyperglycaemic milieu increases the levels of oxidative stress and apoptotic processes during embryogenesis, thus contributing towards the development of birth anomalies [2,19]. Environmental stressors applied early in development can reprogramme the transcriptome of an individual through epigenetic regulation, thereby predisposing them to diseases in adulthood. Specifically, it has been found that SGA and LGA offspring are more likely to develop disorders such as T2DM, obesity, hypertension and coronary artery disease in adulthood [16,20–24]. Research into the long-term effects that maternal diabetes has on offspring health has now indicated a similar link to cardiometabolic diseases in adulthood [25–27]. It is theorised that reprogramming of the genome in response to *in utero* stressors, such as maternal hyperglycaemia, is carried out through epigenetic changes in the genome, which alter gene expression through chromatin structural modifications and changes in the expression levels of non-coding RNAs such as miRNAs [22]. These modifications likely allow the fetus to adjust to an unfavourable intrauterine environment but when these changes are ill-matched to the future post-natal environment, problems can arise [21]. At present, it is not possible to predict which pregnancies complicated by maternal diabetes will result in short- or long-term complications for the fetus, and there are no treatments for LGA or SGA except for early delivery, which in itself can cause complications for the baby [28].

Continuous glucose monitoring (CGM), insulin pumps, sensor-augmented pump therapy, closed-loop systems and metformin therapy have all been employed as contemporary treatment strategies to regulate maternal glucose levels and health outcomes in pregnancies complicated by maternal diabetes [29–32]. However, despite apparently well-managed maternal glucose levels, the prevalence of abnormal fetal growth remains high in pregnancies affected by PGDM and GDM, with LGA occurring in ∼10% of treated GDM, ∼25% of treated T2DM and >50% of treated T1DM pregnancies [33–38]. This suggests that in addition to glucose, other factors may play a role in altering fetal development in diabetic pregnancies.

The placenta is a key regulator of fetal development and is core to the developmental origins of health and disease (DOHaD) hypothesis, where it is premised that suboptimal *in utero* exposures are associated with disease onset later in life [39]. Not only does the placenta maintain an optimal *in utero* environment throughout gestation, but its development invokes direct consequential effects on fetal growth and disease predisposition. As such, understanding how the placenta responds to stressors associated with maternal diabetes may help elucidate the mechanisms that influence fetal programming and offspring health outcomes later in life [40].

## The feto-placental unit

The human placenta is a transient organ that performs essential immunological and endocrine functions which help maintain gestation. Being at the interface between maternal and fetal circulations, it functions to exchange oxygen, carbon dioxide, nutrients and water between the maternal-fetal bloodstreams [41]. Maternal–fetal nutrient transfer capacity of the placenta during gestation has a profound impact on fetal growth, where fetal size is predominantly positively correlated to placental size, function and levels of nutrient transfer [41].

To meet the growing metabolic demands of the fetus and thus allow optimal nutrient transport between the maternal and fetal circulations, placental development and function is closely regulated throughout gestation. Upon implantation, extravillous trophoblasts (EVTs) invade the underlying uterine wall to anchor the placenta and remodel uterine spiral arteries, to enable the perfusion of the placenta with maternal blood [42]. Cytotrophoblasts also differentiate into the multinucleated syncytiotrophoblast which forms the continuous outermost layer of the chorionic villi, termed the syncytium. Throughout gestation, the syncytium expands by the continuous proliferation, differentiation and fusion of the underlying cytotrophoblast populations of the placenta, where material is continuously shed into the maternal circulation [42,43]. These processes are regulated by growth factors, including insulin-like growth factors (IGFs), intracellular signalling cascades, mitochondrial respiration and microRNAs (miRNAs) [44–48].In parallel, vasculogenesis and angiogenesis occur in the villous core to ensure the development of the placental vascular networks; whilst the precise mechanisms responsible for this remain to be established, numerous growth factors including vascular endothelial growth factor (VEGF) are essential regulators [49].

In the term placenta, the syncytiotrophoblast layer, which is in direct contact with maternal blood in the intervillous space, can reach approximately 12 m^2^, enabling a large surface area for exchange of nutrients and gases [50]. Oxygen and nutrients flow through the microvillous membrane (MVM) of the syncytiotrophoblast into the villus core, which is made up of mesenchymal cells and fetal capillaries, where they enter the fetal bloodstream and are transported to the fetus via the umbilical vein [51,52]. Many macro- and micro-nutrients are transported across the placenta, including glucose, fatty acids, amino-acids and folate. GLUT proteins, otherwise known as glucose transporters, located at the MVM and basal plasma membrane (BM) of the syncytium [41], are key for transporting glucose from maternal to fetal circulations via facilitated diffusion [53]. Fatty acid transport proteins (FATPs) are located on the MVM and BM, along with fatty acid binding proteins (FABPs) in the syncytial cytoplasm, which facilitate fatty acid transport across the placental barrier [54,55]. Similarly, amino acid transport proteins such as sodium-coupled neutral amino acid transporters (SNATs), expressed by the syncytiotrophoblast, facilitate the active transport of small non-essential amino acids such as alanine, glycine and serine into the fetal bloodstream [52,54].

Sex differences are observed in the placenta during early gestation, which are then maintained at term and linked to sex-dependent distinctions in adult tissues [56]. Interestingly, it has been shown that males dedicate more resources for fetal growth rather than placental growth, thus making their placentae smaller than females [57]. This suggests that male placentae are more vulnerable to adverse maternal environmental stimuli during gestation [57]. Indeed, given the angioarchitecture of the placenta where it is in constant contact with the maternal circulation, the placenta is heavily susceptible to environmental stressors found in the maternal circulation during pregnancy, such as the diabetic milieu in pregnancies complicated by maternal diabetes [58]. It is thought that such contact with hyperglycaemia and inflammatory cytokines interfere with the normal development of the placenta, leading to altered placental morphology [59]. These alterations to the placenta in pregnancies complicated by maternal diabetes affect its ability to facilitate the transfer of nutrients to the growing fetus, potentially resulting in pathologies such as aberrant fetal growth disorders [59,60]. Furthermore, given that fetal heart development is closely interlinked to placental development [61], it is possible that the impact of maternal diabetes on rates of congenital heart defects and long-term cardiovascular health of the offspring are also attributed to alterations in the placenta.

## Placental development in pregnancies complicated by maternal diabetes

The increasing prevalence of maternal hyperglycaemia during pregnancy has given rise to extensive research on the effects of diabetes on placental development and fetal health. It is already established that altered fetal growth in pregnancies complicated by maternal diabetes is associated with altered placental development [62]. Generally, diabetic pregnancies present with alterations in placental villous maturity, angiogenesis and placental weight [62,63]. Not only does the placenta adapt histologically and molecularly with PGDM and GDM, but these morphological changes also contribute towards altered uteroplacental blood flow which in turn impact fetal nutrient and oxygen supply. Indeed, uteroplacental flow adaptions have been associated with altered fetal growth, and T1DM, T2DM and GDM pregnancies all demonstrate placental hallmarks which contribute towards feto-placental malperfusion [64–71]. To date, most studies reporting the impact of maternal diabetes on the placenta have focussed on GDM. However, even in the limited studies available for T1DM and T2DM pregnancies, it is clear that different types of diabetes exert distinct phenotypic differences on the placenta. As such, it is also important to consider the effect of both GDM and PGDM on placental development.

### Gestational diabetes mellitus

GDM is defined as maternal glucose intolerance that is first identified during pregnancy and is typically diagnosed through an oral glucose tolerance test (OGTT) at weeks 24–28 gestation [72,73]. The diagnostic criteria and degree of hyperglycaemia in individuals with GDM can vary widely, resulting in broad clinical manifestations of GDM. Pancreatic β-cell dysfunction and insulin resistance are features of GDM and in some cases, it is thought that these hallmarks may already be underlying prior to conception, and are exacerbated during the maternal metabolic adaptations that occur in pregnancy [73]. Interestingly, it has been reported that the risk and severity of GDM is increased in pregnancies carrying a male fetus, where maternal blood glucose levels at OGTT are increased and β-cell function is reduced [74]. Controversy remains as to whether perinatal outcomes are less favourable for male or female offspring from pregnancies complicated by GDM [75–78]. Lifestyle interventions such as exercise and diet modifications are the main treatment for GDM. However, metformin and insulin are also used if maternal glycaemic levels do not improve. Glibenclamide is used rarely [73].

In contrast with uncomplicated pregnancies, GDM manifests with placental histological adaptions such as villous immaturity, villous oedema, decidual vasculopathy, chorangiosis, fibromuscular sclerosis, villous agglutination, retroplacental hemorrhage, altered fibrinoid necrosis, increased volume of intervillous space, terminal villous volume and surface area, as well as increased syncytiotrophoblast turnover and knotting [79–83].These changes, along with altered placental amino acid and lipid transport result in aberrant feto-placental nutrient transfer [79]. Altered DNA methylation patterns and differentially expressed genes associated with cell death and activation, immune response and organ development have also been characterised in the placentae of those with GDM compared with uncomplicated pregnancies [84–86]. Other hallmarks of GDM placentae include altered oxidative stress and autophagy, mitochondrial dysfunction, placental macrophage (Hofbauer cell) accumulation and increased expression of inflammatory factors [87–93].

GDM is associated with a state of chronic low-grade placental inflammation, where proinflammatory cytokine expression is demonstrated to be sex-dependent [94]. Indeed, immune related pathways are shown to be altered in the placenta of male pregnancies complicated by GDM, further suggesting that the effect of the maternal diabetic milieu on placental immune pathways may be sex-specific [95]. GDM and offspring sex have also been identified to impact placental mitochondrial biogenesis, which could potentially result in male offspring being at increased risk of developing metabolic diseases during adulthood compared with females [96]. A sexually dimorphic effect has also been reported in placental amino acid metabolism in GDM pregnancies. As altered amino acid metabolism is associated with preeclampsia, intrauterine growth restriction and altered fetal brain development, this suggests that male and female offspring have a varied predisposition to gestational complications in GDM pregnancies [95]. A similar sex-specific effect has been identified in placental protein glycosylation in GDM, in that male placental O-GlcNAc transferase (OGT) expression is reduced compared with females, which may impact placental hormonal production [97].

### Type 1 diabetes mellitus

T1DM is characterised by pancreatic β-cell destruction, resulting in insulin insufficiency and hyperglycaemia. For most people, this pancreatic destruction is driven by autoimmunity. Insulin therapy is the main treatment for T1DM, including contemporary strategies such as CGM, insulin pumps and closed-loop systems. Although often diagnosed during childhood or adolescence, T1DM can also manifest later in life [98].

Various studies have demonstrated that T1DM pregnancies present with unique placental hallmarks associated with altered vascularisation which have not been reported in the placentae of those with GDM. These alterations include increased vascular leakiness, and increased capillary diameter, branching and capillary wall elongation, resulting in a higher villous volume and surface area [58,99,100]. Accelerated villous maturation is also more prominent in T1DM placentae compared with GDM [101]. Interestingly, placental GLUT-1 protein expression is also higher in T1DM pregnancies compared with GDM and has been positively correlated to fetal weight [102], suggesting altered glucose transfer. Although placentae of T1DM do not manifest as many lipid modifications as GDM, placental glycosylation and acylation pathways are more pronounced [103]. In contrast with GDM, people with T1DM manifest hyperglycaemia prior to conception and during early pregnancy. As such, these findings suggest that glucose and lipid metabolism, as well as glycosylation and acylation pathways in the placenta may be more sensitive to diabetic stimuli during early gestation rather than later in pregnancy. This may also be the case for placental vascularisation and villous maturation, where individuals with GDM do not present with these placental morphologies. Although it is not fully established whether these hallmarks are altered during early pregnancy/first trimester placenta of people with T1DM, it has been demonstrated that hyperglycaemia reduces first-trimester trophoblast turnover in T1DM placentae [104]. Further studies are needed to elucidate the broader effects of the T1DM diabetic milieu on the placenta in early pregnancy.

It is thought that the duration of T1DM may also be associated with adverse outcomes. Indeed, impaired placental extracellular matrix remodelling is a feature of T1DM pregnancies which has been shown to be augmented in longer-term T1DM mouse models [105–107]. Another feature of T1DM pregnancies is altered placental cellular stress [108]. Markedly reduced placental aerobic respiration activity and up-regulated hydrogen peroxide levels are observed in individuals with T1DM compared with BMI-matched normoglycaemic people [109]. However, T1DM placentae illustrate protective mechanisms against oxidative stress compared with GDM, where there is increased glutathione peroxidase activity, higher abundance of reduced glutathione and lower levels of oxidised glutathione [110]. Perhaps these protective mechanisms in T1DM may be as a consequence of longer-term adaptations to the diabetic milieu from conception, compared with those with GDM who are exposed to a diabetic environment for a shorter duration later in pregnancy and thus have a brief time-frame to develop protective adaptations in the placenta. This interpretation may also explain why placental infarcts were observed to be less abundant in T1DM pregnancies compared with GDM [64]. However, T2DM pregnancies also manifest with maternal hyperglycaemia as early as conception and interestingly, more infarcts were observed in the placentae of people with T2DM than T1DM [111]. It is possible that this finding may reflect study design, where pregnancy loss was not captured and only surviving pregnancies with fewer abnormalities were included in the study. T1DM is associated with more extreme first-trimester hyperglycaemia, congenital abnormalities and pregnancy loss than T2DM, and therefore perhaps this led to the collection of healthier placental samples of T1DM pregnancies compared with T2DM [111].

Another placental feature of T1DM pregnancies is heightened baseline vascular tone. It is thought that this may be a result of increased nitric oxide (NO) pathway activity in diabetes, unrelated to insulin levels [112]. Altered uterine NO-mediated vasodilation has been demonstrated in pregnant mice with GDM, where augmented superoxide levels may promote increased NO scavenging [113,114]. Aberrant adenosine-stimulated vasocontractility has also been identified in the feto-placental vasculature of GDM and T1DM pregnancies [115]. However, further research is needed to investigate whether altered placental NO activity applies for GDM and T2DM pregnancies or is unique to T1DM. Moreover, systemic endothelial dysfunction has been associated with PGDM and GDM pregnancies [116,117].

Despite intensive strategies to achieve normoglycaemia in pregnant individuals with T1DM, these pregnancies still manifest distinct placental hallmarks contributing towards adverse fetal outcomes compared with GDM and healthy, uncomplicated pregnancies. As such, more studies are needed to explore the mechanisms contributing towards altered placental morphologies in T1DM pregnancies.

### Type 2 diabetes mellitus

Another form of PGDM is T2DM; a heterogenous condition characterised by hyperglycaemia that is driven by insulin resistance and/or impaired pancreatic β-cell insulin secretion. This type of diabetes presents with varying underlying pathophysiology but is strongly associated with adiposity and a background of skeletal muscle, liver and adipose tissue insulin resistance [118]. Lifestyle interventions such as exercise, diet changes and weight loss are the main initial treatment strategy for T2DM. However, multiple oral hypoglycaemic agents and injectables such as insulin and glucagon-like peptide-1 (GLP-1) agonists are also commonly required [118,119]. Although traditionally, T2DM occurred with advancing age, it is increasingly being diagnosed in children and young adults; this is known as early onset T2DM (EOT2D). This is a concern as it is a more severe condition if diagnosed <40 years-of-age and associated with greater risk of complications. T2DM is now far more common than T1DM in women of child-bearing age [118–120].

T2DM pregnancies are characterised by up-regulated placental glucose, amino acid and fatty acid transporter expression compared with BMI-matched normoglycaemic pregnancies [121,122]. This highly suggests that feto-placental nutrient transfer and metabolism are altered in T2DM. With T2DM being a heterogenous condition that is predominantly associated with adiposity, it is possible that the altered placental nutrient transfer observed in these individuals may reflect maternal nutrient excess that is associated with T2DM pregnancies. As such, it is reasonable to assume that pathological placental development in individuals with T2DM may be driven by metabolic disturbances of various metabolic tissues rather than pancreatic dysfunction exclusively. Indeed, lipoperoxidation is another placental feature of T2DM pregnancies, as well as placental calcification which is mostly identified in T2DM compared with T1DM and GDM pregnancies [122,123]. These placental hallmarks have been associated with maternal adiposity in mice, where increased placental labyrinth lipid peroxidation and calcification have been identified in male offspring of obese dams [124]. Moreover, placental calcification has been identified as a predictor of suboptimal uteroplacental flow [125], and this is further evidenced by the increased prevalence of decidual vasculopathy in T2DM compared with GDM pregnancies [64].

Maternal obesity has also been demonstrated to influence placental labyrinth adaptations to cellular stress [124]. It is possible that this could be a response to the altered placental aerobic respiratory activity that is observed in people with T2DM [109]. Inflammation is another feature mostly associated with T2DM pregnancies compared with T1DM and GDM [123]. This hallmark is consistent with placental features of pregnancies complicated by maternal obesity, where it is suggested that increased inflammation may be a response to exacerbated cellular oxidative stress and altered metabolism [126].

Moreover, similarly to T1DM, accelerated placental villous maturation has also been identified in T2DM pregnancies [101,102]. Disorders of villous maturity are associated with fetal death [127] and it has been demonstrated that PGDM increases the risk of stillbirth, where obesity can amplify this risk [128]. These findings, along with the altered placental glucose metabolism identified exclusively in PGDM pregnancies [102,121], further suggest that placental glucose transport and villous maturation may be most susceptible to the maternal diabetic milieu at earlier stages of pregnancy. Additional studies on first trimester placental tissue are required to validate the effects of PGDM on adverse fetal outcomes compared with GDM pregnancies.

### Factors contributing towards placental pathology in diabetes

*In utero* hyperglycaemia is inherent to GDM, T1DM and T2DM pregnancies. However, each diabetes type demonstrates a distinct set of placental features. This is unsurprising given the variance in clinical manifestations and pathophysiological characteristics belonging to each type and thus suggests that factors other than glucose may contribute towards placental phenotypic adaptations in diabetes. This may include hypoglycaemic agents such as metformin, which has been demonstrated to alter placental development and function [29,129,130]. This is further supported by studies showing that individuals with well-managed glucose levels still manifest with pathological placental hallmarks [131–133]. These changes in the placenta could potentially be explained by the increasing evidence demonstrating that in addition to glycaemic control, fetal sex, maternal weight, ethnicity and the underlying pathophysiology and duration of the diabetes types can all contribute towards distinct pathological hallmarks [63,107]. There are limited studies investigating the possible mechanisms responsible for the impact of T1DM and T2DM on the placenta and fetus, however, clear evidence shows that miRNAs are key regulators of normal placental development and are mediating factors contributing towards placental pathology and associated adverse outcomes in pregnancies complicated by GDM ([134–138], Tables 1-3).

**Table 1:** Altered placental miRNA regulation and their known targets and functional outcomes in pregnancies complicated by maternal diabetes

Diabetes type	miRNA regulation	Model	Target	Functional outcome	Reference
GDM	↓ miR-29b	Human placental tissue and HTR-8/SVneo cells	↑ HIF3A	↑ cell migration and invasion	[210]
GDM	↓ miR-143	Human placental tissue and primary syncytiotrophoblast	↓ PPARγ ↓ PGC1α ↑ hPL ↑ GLUT1 ↑ mTOR	↓ mitochondrial respiration	[211]
GDM	↓ miR-21	Human placental tissue and HTR-8/Svneo cells	↑ PPARα	↓ cell growth and infiltration	[212]
GDM	↑ miR-518d	Human placental tissue and HEK-293T cells	↓ PPARα	Altered fatty acid and glucose metabolism	[213]
GDM	↑ miR-98	Human placental tissue, JEG-3 and HEK-293T cells	↓ MeCP2 ↓ TRPC3	↑ global DNA methylation ↓ insulin-mediated- uptake of glucose	[214]
GDM	↓ miR-138-5p	Human placental tissue and HTR-8/Svneo cells	↑ TBL1X	↑ cell proliferation and placental growth	[215]
GDM	↓ miR-9 ↓ miR-22	Human placental tissue, primary syncytiotrophoblast, HEK-293T and HTR-8/Svneo cells	↑ GLUT1 ↑ HK2	↑ glucose uptake, lactate secretion and cell viability ↓ apoptosis	[216]
GDM	↑ miR-140-3p	Human placental tissue and umbilical vein endothelial cells, HEK-293T and HTR-8/Svneo cells	↓ IR-α ↓ IGFR1	Defective insulin receptor signalling	[217]
GDM	↓ miR-132	Human placental tissue and BeWo and HTR-8/Svneo cells	PTEN	↓ cell proliferation	[218]
GDM	↓ miR-6795-5p	Human placental tissue, HEK-293T and HTR-8/Svneo cells	PTPN1	Altered insulin signalling, cell growth and glucose metabolism	[219]
GDM	↑ miR-136	Human placental tissue, BeWo and HTR-8/Svneo cells	↓ E2F1	↓ cell proliferation	[220]
GDM	↓ miR-345-3p	Human placental tissue, HEK-293T and HTR-8/Svneo cells	↑ BAK1	↑ apoptosis ↓ proliferation and migration	[221]
GDM	↑ miR-95 ↑ miR-548am ↓ miR-1246	Human placental tissue	↓ GLUT1 ↓ GLUT3 ↓ GLUT4	Aberrant insulin signalling pathway	[222]
GDM	↓ miR-22 ↓ miR-372	Human placental tissue and HTR-8/Svneo cells	↓ GLUT4	Aberrant insulin signalling pathway	[223]
GDM	↓ miR-30d-5p	Human placental tissue and HTR-8/Svneo cells	↑ RAB8A	↑ cell proliferation, migration, invasion and glucose uptake	[163]
GDM	↑ miR-1323	Human HTR-8/Svneo and BeWo cells	↓ TP53INP1	↓ cell viability	[224]
GDM	↑ miR-657	Human placental mononuclear macrophages and THP-1 cells	↓ FAM46C	↑ cell proliferation, migration and polarisation towards M1 phenotype	[225]
GDM	↑ miR-199a	Human placental tissue and JEG-3 cells	↓ MeCP2 ↓ TRPC3	Altered methylation patterns and glucose metabolism	[226]
GDM	miR-195-5p	Human pulmonary microvascular endothelial cells and mouse placental tissue	↓ VEGFA	↑ endothelial cell dysfunction	[227]
GDM	↓ miR-6869-5p	Human placental mononuclear macrophages and THP-1 cells	↑ PTPRO	↓ cell proliferation, inflammatory response ↑ polarisation towards M2 phenotype	[228]
GDM	↑ miR-101	Human umbilical vein endothelial cells	↓ EZH2	↓ gene transcription	[229]
GDM	↑ miR-134-5p	HTR-8/Svneo and HEK-293T cells	↓ FOXP2	↑ inflammation and apoptosis	[230]
GDM	↑ miR-137	Human umbilical vein endothelial cells, U937 and THP-1 cells	↑ CCL2	↓ cell viability and angiogenesis ↑ inflammatory cytokine secretion, cell activation, monocyte chemotaxis and adhesion	[231]
GDM	↓ miR-9-5p	Human placental tissue and primary syncytiotrophoblast	↑ HK2 ↑ GLUT1 ↑ PFK ↑ LDH	Altered aerobic glycolysis and mitochondrial complex expression	[232]
GDM	↑ miR-195-5p	Human umbilical vein endothelial cells and HEK-293T cells	↓ EZH2	↓ cell proliferation and viability ↑ apoptosis	[233]
GDM	↓ miR-96-5p	Human placental tissue and HTR-8/Svneo cells	-	↓ cell viability	[234]
GDM	↓ miR-193b	HTR-8/Svneo cells	↑ IGFBP5	↑ autophagy and apoptosis	[235]
GDM	↑ miR-34b-3p	Human umbilical vein endothelial cells	↓ PDK1	↓cell viability and migration	[236]
GDM	↑ miR-190b	Human placental tissue, Min6 cells and mouse β-cells	↓ NKX6-1	↓ cell activity, proliferation, islet insulin secretion	[237]
GDM	↑ miR-503	Human placental tissue, INS-1 and HEK-293T cells	↓ mTOR	Pancreatic β–cell dysfunction	[238]
GDM	↑ miR-144 ↓ miR-125b	Human placental tissue	-	Abnormal glucose metabolism	[239]
GDM	↓ miR-96	Human placental tissue, INS-1 and HEK-293T cells	↑ PAK1	↓ insulin secretion and β-cell function	[240]
GDM and T2DM	↓ miR148a-3p ↓ miR29a-3p	Human placental tissue and umbilical vein endothelial cells	AMPKα1 IGFR1 IRS1/2 PPARγ PI3K	Altered insulin signalling and glucose metabolism	[209]
T1DM	*No studies identified*				
T2DM	*No studies identified*				

Abbreviations: AMPK, adenosine monophosphate-activated protein kinase; BAK, BCL-2 homologous antagonist killer; CCL, C-C motif chemokine ligand, E2F1, E2F transcription factor 1; EZH2, enhancer of zeste 2 polycomb repressive complex 2 subunit; FAM46C, family with sequence similarity 46, member C; FOXP, forkhead box protein P; GALNT, polypeptide N-acetylgalactoaminyltransferase; GLUT, glucose transporter; HIF, hypoxia-inducible factor; HK, hexokinase; hPL, human placental lactogen; IGFBP, insulin-like growth factor-binding protein; IGFR, insulin-like growth factor; IR, insulin receptor; IRS, insulin receptor substrate; LDH, lactate dehydrogenase; MeCP, methyl CpG binding protein; mTOR, mammalian target of rapamycin; NKX6-1, NK6 homeobox 1; PAK, p21-activated kinase; PDK, pyruvate dehydrogenase kinase; PFK, phosphofructokinase; PGC, peroxisome proliferator-activated receptor-γ coactivator; PI3K, phosphoinositide 3-kinase; PPAR, peroxisome proliferator-activated receptor; PTEN, phosphatase and tensin homolog; PTPN, protein tyrosine phosphatases, non-receptor type; PTPRO, protein tyrosine phosphatase receptor type O; RAB8A, Ras-related protein Rab-8A; TBL1X, transducin β like 1 X-linked; TP53INP, tumour protein p53-inducible nuclear protein; TRPC, transient receptor potential channel; VEGF, vascular endothelial growth factor.

**Table 2:** Altered maternal circulating miRNAs of placental origin and their known targets and functional outcomes in pregnancies complicated by maternal diabetes

Diabetes type	miRNA Regulation	Source	Target	Functional outcome	Reference
GDM	↑ miR-135a-5p	Maternal plasma EVs (including characterisation of placenta-derived EVs based on PLAP expression)	↑ SIRT1	↑ trophoblast proliferation, invasion and migration	[273]
GDM	↑ miR-130b-3p	Secreted EVs from cultured placental MSCs	↓ ICAM-1	↑ HUVEC proliferation, migration and angiogenesis	[274]
GDM	↓ miR-140-3p ↓ miR-574-3p	Secreted EVs from cultured placental villous explants	↓ VEGF	↑ cell proliferation, migration and tube formation	[275]
GDM	↑ miR-125a-3p ↑ miR-224-5p ↑ miR-584-5p ↑ miR-186-5p ↑ miR-22-3p ↑ miR-99b-5p ↑ miR-433-3p ↑ miR-197-3p ↑ miR423-3p ↓ miR-208a-3p ↓ miR-335-5p ↓ miR-451a ↓ miR-145-3p ↓ miR-369-3p ↓ miR-483-3p ↓ miR-203a-3b ↓ miR-574-3p ↓ miR-144-3p ↓ miR-6795-5p ↓ miR-550a-3-3p ↓ miR-411-5p ↓ miR-550a-3-3p ↓ miR-140-3p	Secreted EVs from cultured placental villous explants	–	↓ primary skeletal muscle cell insulin-stimulated migration and glucose uptake	[276]
GDM	↓ miR-516-5p ↓ miR-517-3p ↓ miR-518-5p ↓ miR-222-3p ↓ miR-16-5p	Maternal urine EVs (including characterisation of placenta-derived EVs based on PLAP expression)	IRS4 GALNT RECK ALG3 AKT3 TIMP3 KIT L2HGDH KI2FC RAP1 HOXC8 PD-L1	Altered insulin signalling, metabolic homeostasis and inflammatory response	[199]
GDM	↑ miR-520h ↑ miR-1323 ↑ miR-136-5p ↑ miR-342-3p	Maternal serum EVs (including characterisation of placenta-derived EVs based on PLAP expression)	↓ AMPK ↓ GLUT2	Altered β-cell insulin secretion, β-oxidation and glucose transport	[200]
T1DM	*No studies identified*.				
T2DM	*No studies identified*.				

Abbreviations: AKT, RAC-gamma serine/threonine-protein kinasel ALG3, asparagine-linked glycosylation protein 3 homolog; AMPK, adenosine monophosphate-activated protein kinase; GALNT, polypeptide N-acetylgalactoaminyltransferase; GLUT, glucose transporter; HOX, homeobox; HUVEC, human umbilical vein endothelial cell; ICAM, intracellular adhesion molecule; IRS, insulin receptor substrate; KI2FC, kinesin family member 2C; KIT, KIT proto-oncogene, receptor tyrosine kinase; L2HGDH, L-2-hydroxyglutarate dehydrogenase; MSC, mesenchymal stem cell; PD-L, programmed death-ligand; PLAP, placental alkaline phosphatase; RAP, Ras-related protein; RECK, reversion inducing cysteine rich protein with kazal motifs; SIRT, Sirtuin; TIMP3, tissue inhibitor of metalloproteinase-3; VEGF, vascular endothelial growth factor.

**Table 3:** miRNAs associated with altered fetal growth in pregnancies complicated by maternal diabetes

Model	Source	miRNA regulation	Functional outcome/target	Fetal growth outcome	Reference
GDM	Human placental tissue	↑ miR-508-3p ↓ miR-27a ↓ miR-9 ↓ miR-137 ↓ miR-92a ↓ miR-33a ↓ miR-30d ↓ miR-362-5p ↓ miR-502-5p	EGFR signalling	Macrosomia	[283]
GDM/T2DM	Human maternal serum and placental tissue	↑ miR-16/↓ miR-16	CUL4A SMAD1 EGFR ACTB RRP12 DAB2	Macrosomia/SGA	[284,285]
GDM	Human maternal plasma and placental tissue	↓ miR-517a	↑ IGF-1 and trophoblast proliferation	Macrosomia	[286]
GDM and T2DM	Human placental tissue	↓ miR-126-3p	IRS1 PI3K	Lower birth weight	[209]

Abbreviations: ACTB, β-actin; CUL4A, Cullin 4A; DAB2, Disabled-2; EGFR, epidermal growth factor receptor; IGF-1, insulin-like growth factor 1; IRS1, insulin receptor substrate 1; PI3K, phosphatidylinositol 3-kinase; RRP12, ribosomal RNA processing 12 homolog; SMAD1, small body size and mothers against decapentaplegic family 1

## miRNAs in pregnancy

### microRNA biogenesis

microRNAs are essential for most cellular and biological processes, including regulating placental development [134,139]. miRNAs are non-coding RNAs that are temporally expressed at different developmental timepoints. They modulate gene network expression post-transcriptionally, either by suppressing mRNA translation via signalling mRNA for degradation or deadenylation, or by stimulating gene expression via relief of repression [140–142]. With an average size of 22–23 bp, these single-stranded miRNAs are highly conserved structures which are produced from a tightly regulated process. First a hairpin loop structure, known as primary(pri)-miRNA is transcribed from genic or intergenic (known as mirtrons) regions in the nucleus. These pri-miRNA are then processed to 60–70 nucleotide-long precursor(pre-)miRNA and then 22–23 nucleotide mature miRNA molecules through a sequence of events involving the endonucleases, Drosha and Dicer. Following the processing of pre-miRNA, it was previously thought that one strand of the miRNA duplex was degraded, leaving only one functionally active mature strand. However, it has now become apparent that both strands of the miRNA duplex are functionally active. As such, this has coined the -3p and -5p nomenclatures used to describe miRNAs, in order to differentiate the mature miRNAs deriving from the 3′ and 5′ terminals of the pre-miRNA hairpin structure [143]. Mature, functionally active miRNA strands then bind the 3′-untranslated region (UTR), or in some instances, 5-UTR, of target mRNA to induce translational repression or mRNA degradation; reviewed in [139,141]. Multiple miRNAs work simultaneously to regulate broad gene networks, resulting in pleiotropic downstream effects in the cell or tissue in which they reside. Interestingly, many miRNAs are temporally synthesised in a tissue-specific manner, including the placenta whereby distinct expression profiles are found at different stages of gestation [134–136], suggesting that miRNAs may play specific roles in the placenta. Indeed, several studies have shown that miRNAs are key regulators of placental development and function [134].

### microRNAs in the placenta

Some of the most compelling evidence for a role of miRNAs in the placenta is evident from evolutionary studies. Recently, a group of 13 miRNA gene families have been shown to originate early in placental mammal evolution, suggesting a key role in processes specific to placental mammals [137]. Whilst these miRNAs are expressed in many cells and tissues, evidence supporting this comes from a recent study showing that within the endometrium, some of these evolutionary conserved miRNAs have an important role in regulating the initial stages of implantation [144]. Given their expression in the human placenta, it is likely that these miRNAs also play key roles in the placenta. Other evidence for a strong link between the evolutionary conservation of miRNAs across various placental mammals comes from work demonstrating that some miRNA encoding genes arise from different evolutionary chromosomal clusters and are significantly or exclusively expressed in the placenta of various mammalian species [138].

The chromosome 19 miRNA cluster (C19MC) is a key maternally imprinted, primate specific, cluster found within the human placenta, where 46 genes encode 59 mature, placental-specific specific miRNAs [134,135,145,146]. The eutherian specific, paternally imprinted chromosome 14 miRNA cluster (C14MC) is another key cluster consisting of 52 miRNA genes which encodes 84 mature miRNAs that are mostly exclusively expressed in the placenta [147–150]. This cluster is divided into genomic regions known as the miR-127/miR-136 and miR-379/miR-410 clusters [151]. The marked or exclusive expression of these miRNA clusters within the placenta suggests that they have key functional relevance in this organ. Indeed, miRNAs originating from C19MC are the predominant miRNAs found in term human trophoblasts and are key in regulating trophoblastic mRNA and protein profiles to maintain cellular homeostasis [145]. Placental targets of C19MC miRNAs have also been mapped to functions associated with DNA binding, protein phosphorylation, cytokine response, oxidative stress and regulation of growth and apoptosis [146]. The miR-371-3 cluster, located downstream to C19MC, is also primarily expressed in placental tissue and is involved in regulating cellular proliferation and apoptosis [152,153]. The role of C14MC miRNAs in pregnancy is yet to be fully established; however, the miR-127/miR-136 cluster has been shown to be involved in fetal capillary development and the miR-379/miR-410 cluster has been demonstrated to influence trophoblast proliferation and migration [147,151]. Another cluster involved in placental development is miR-17/92. This cluster is not placenta-specific but consists of six miRNAs (miR-17, miR-18a, miR-19a, miR-19b, miR-20a and miR-92a) which mediate key placental growth processes such as angiogenesis, trophoblast proliferation, spiral artery remodelling and cell cycle regulation [154,155]. Evidence also points towards miR-17/92 and its paralog cluster, miR-106a-363, playing a role in trophoblast differentiation through the regulation of estrogen receptor α (ERα) [156].

It has been established that the placental miRNA atlas is sexually dimorphic [157]. Whilst there is a clear evolutionarily conserved role for some placental miRNAs, more than 2000 mature miRNAs have been detected in the human placenta and the vast majority of these are highly conserved across different cells and tissues, likely due to their roles in key physiological or homeostatic processes. Indeed, the top four most abundant placental miRNAs (miR-30d-5p, miR-100-5p, miR-143-4p and miR-21-5p) play significant roles in tissues beyond the placenta [48,158–162]. miR-30d has been associated with cancer progression and cardiac hypertrophy [163,164]. miR-100-5p is known to regulate skeletal muscle myogenesis [165] and miR-143-3p has been demonstrated to regulate vascular smooth muscle differentiation and modify autophagy in endometrial stromal cells [166,167]. miR-21 is highly conserved and is almost ubiquitously expressed, where its abundance has been associated with HTR8/SVneo cell proliferation and pregnancies complicated by preeclampsia [168,169]. It has also been shown that miR-21 plays a key role in epithelial–mesenchymal transition [170]. There are other various regulatory miRNAs which are key for placental development [171], some of which include; let-7a and miR-145, which regulate trophoblast proliferation, and vascular development and cell turnover of other tissues [48,158–162]; miR-96-5p which regulates proliferation and migration of trophoblasts as well as in vascular smooth muscle cells [172,173]; miR-29a which regulates muscle and skeletal function and homeostasis, immune system modulation and haematopoiesis of various tissues [174,175]; and miR-125b which regulates trophoblast migration and invasion, as well as playing a role in mitochondrial biogenesis and adipocyte development and function [176,177]. Given the abundance of other miRNAs in the placenta and evidence that several are altered in pregnancy complications such as fetal growth restriction, other yet unreported roles for miRNAs is likely. Indeed miR-16, miR-21 and miR-199a are examples of this. These miRNAs are associated with fetal growth and whilst their functional roles in the placenta remain to be established, these miRNAs regulate insulin sensitivity and glucose metabolism in other cells and tissues [178–183]. Exploring these roles in the placenta would further our understanding of the currently unreported roles of various miRNAs in pregnancy.

### Circulating microRNAs in pregnancy

In addition to considering the role of miRNAs that are detected in the placenta, the role of miRNAs in the circulation should also be considered. Indeed, sexually dimorphic patterns have been identified for maternal circulating miRNAs in pregnancies complicated by altered fetal growth [184]. Whilst miRNAs are transcribed and functionally active in their tissue of origin, they can be released into circulation encapsulated in extracellular vesicles (EVs). EVs are produced from all cells and tissues and can be subcategorised according to size, density, molecular composition or cellular origin into the following classes; small EVs (typically <200 nm in diameter) and large EVs (typically >200 nm in diameter) [185–187]. EVs contain DNA, miRNAs, mRNAs, proteins and lipids, with their cargo reflecting phenotypic hallmarks of their cell of origin [188,189]. As such, EVs and their cargo demonstrate potential as biomarkers for different pathological conditions. However, other ‘hormonal-like’ roles for EVs are also emerging. When released from cells, EVs can be transported into local and distal cells via a variety of mechanisms including clathrin-mediated endocytosis, membrane fusion, macropinocytosis and phagocytosis [190] and thus can influence the transcriptome of target cells via their miRNA cargo. As such, this EV-mediated transport in the human circulation allows systemic bidirectional interorgan cross-talk via miRNA regulation [188].

With regards to the placenta, its production of EVs has been long established, following the discovery that syncytiotrophoblast sheds microparticles (0.2–2 µm) that are capable of immunoregulation of circulating monocytes [191–193]. More recently, it has been reported that the concentration of EVs in the maternal circulation is increased in pregnancy, and that placental-derived EVs play a key role in regulating maternal glucose homeostasis [194–196]. Interestingly, EV-labelling techniques in *in vivo* murine models have shown that placental EVs can also interact with various maternal cells and tissues, including endothelial and immune cells, lung, kidney and liver [194]. This suggests that placenta-derived EVs may also have other important roles in regulating maternal homeostasis during pregnancy.

Whilst the specific EV cargo involved in these interactions have not been fully elucidated, several studies suggest that they are likely attributed to miRNAs. Indeed, the presence of trophoblast-specific C19MC and non-C19MC miRNAs in the maternal circulation during pregnancy has been reported [147,197,198] and placenta-derived EV-miRNA profiles have been shown to be altered with gestational age [199,200]. Functional roles for placental derived EV miRNAs have also been reported. EV-encompassed C19MC and miR-17-92 cluster miRNAs have been shown to play immunomodulatory roles during pregnancy by influencing events in maternal immune cells [145,201–203].

While the concept of feto-maternal signalling via EVs and their miRNA cargo is well regarded in the literature, evidence is also emerging for a role of EVs and their cargo in maternal–placental communication. Holder et al.’s [2016] visualisation of the internalisation of PKH labelled maternal macrophage-derived EVs by the placenta was one of the first to show that EV-mediated communication between maternal cells/tissues and the placenta is bidirectional [204]. The functional consequence of maternal–placental EV communication has also been established, where maternal macrophage EVs modulate placental cytokine production [204] and maternal adipose tissue EVs influence placental glucose metabolism by altering genes involved in glycolysis and gluconeogenesis [188]. Whilst the EV cargo responsible for exerting these maternal–placental effects of EVs has yet to be established, there are reports that miRNAs released from maternal organs can traffic into placental and fetal tissues to influence feto-placental development [188,197,205]. This suggests that the maternal metabolic state and environment may impact on placental function and fetal growth via EV-miRNAs. Recent work within our group also supports this hypothesis [184,206,207]. It is therefore possible that whilst many miRNAs are produced by the placenta, other mature miRNAs present in the placenta are a consequence of being transported to the placenta from maternal (or fetal) circulations, and that these may be altered in pathological conditions.

## microRNAs in pregnancies complicated by maternal diabetes

### Placental miRNAs

In pregnancies complicated by maternal diabetes, there is an altered placental miRNA profile compared with healthy pregnancy (Table 1). Whilst the mechanisms through which a diabetic environment alters placental miRNA levels remain to be elucidated, it is clear that miRNAs have an integral role in feto-placental development and that they may contribute to adverse outcomes in these pregnancies. miRNAs elicit functional changes in target expression through DNA methylation-associated mechanisms which represses messenger RNA transcription. In turn, miRNA-encoding genes and some miRNAs themselves may also be methylated to regulate miRNA expression [208]. It has been postulated that these methylation patterns may influence miRNA regulation in response to certain treatments. As such, this shows that miRNAs have a significant role in multifaceted epigenetic events which may play important roles in health and disease functional outcomes [208].

**Figure 1:**
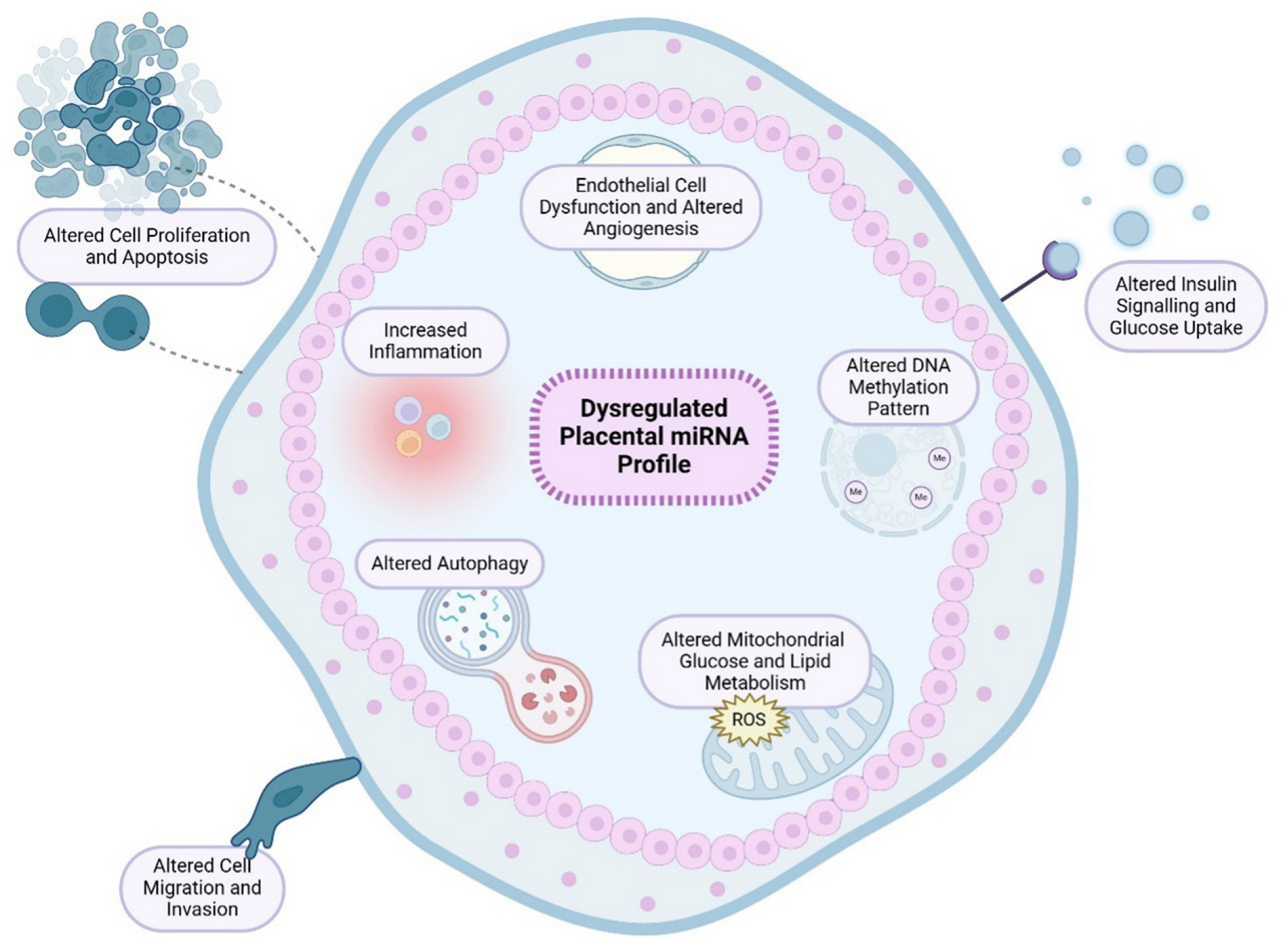
Effect of maternal diabetes on placental miRNA profile and functional outcomes Pregnancies complicated by maternal diabetes are associated with a dysregulated placental miRNA and proteomic profile. As a result, studies have identified maternal diabetes leads to alterations in placental growth, insulin signalling and glucose uptake, epigenetic regulation, cell migration and invasion, vascular development, inflammation, autophagy and mitochondrial metabolism. *Created using Biorender.com*.

Using various models, it has been established that miRNAs that have been identified to be altered by maternal diabetes in placental tissue have many overlapping targets; sharing functional hallmarks associated with placental growth, insulin signalling, glucose metabolism, inflammation and vascular development [163,209–240] (Table 1 and Figure 1). Indeed, among these dysregulated miRNAs is miR-9, which has also demonstrated to regulate HUVEC angiogenesis, proliferation, migration and invasion [241]. Additionally, people with GDM not only demonstrate dysregulated miR-222 expression in their placental tissue but also in their adipose tissue [216,223,242]. Interestingly, this miRNA is a key regulator of ERα expression in estrogen-induced insulin resistance [242]. miR-503 is another dysregulated miRNA found in the placenta of individuals with GDM [238]. This miRNA is involved in controlling inflammation-mediated angiogenesis, where its expression in HUVECs is up-regulated with high glucose [238,243–245]. As such, these findings provide potential connections between the maternal diabetic environment and placental vascular dysfunction. In addition to altered vascular function, various other hallmarks in the GDM placenta are known to be closely associated with altered fetal growth and therefore suggest possible mechanisms linking maternal diabetes to altered fetal growth [246–250].

GDM has also been associated with sexually dimorphic placental miRNA expression. In female pregnancies, these miRNAs are associated with pathways involving proteoglycans in cancer, protein processing in endoplasmic reticulum and signalling cascades which regulate vascular development, apoptosis and proliferation [57,251]. In contrast, male GDM pregnancies only demonstrate altered placental miRNA expression that is associated with extracellular matrix–receptor interaction. As such, this suggests that the GDM environment has a more pronounced impact on the miRNA placental regulation of female pregnancies compared with male pregnancies [57,251].

### Aetiology of altered miRNA profiles in the placenta

The aetiology of altered placental miRNA expression in maternal diabetes remains unclear. One possible mechanism is that maternal diabetes may be characterised by aberrant miRNA biogenesis. Previous research shows placental Dicer dysregulation leads to changes in trophoblast proliferation, suggesting Dicer-dependent miRNAs are involved in regulating placental development [252]. Similar findings have been observed in the placentae of people with GDM, where Dicer, Drosha and DGCR8 expression are found to be altered, leading to changes in miRNA biogenesis [253]. Nonetheless, given that the majority of miRNAs are Dicer, Drosha and DGCR8-dependent miRNAs, this is unlikely to explain why only specific miRNAs are altered in the placenta of individuals with maternal diabetes. An alternative hypothesis is that high glucose levels, or other components of the diabetic environment, may be directly altering miRNA expression in the placenta and various maternal tissues. In turn, miRNAs may be important mediators between glucose fluctuations and downstream functional effects in the placenta. Of interest, many of the miRNAs with key roles in feto-placental growth and regulation are known to be glucose sensitive, which is an important consideration in pregnancies complicated by maternal diabetes [173,175,177,179,180,254–257]. Not all studies report the status of maternal glucose control, however, variation in the placental hallmarks observed in T1DM, T2DM and GDM pregnancies suggests factors other than glucose may be involved. This is further evidenced by a previous report demonstrating that people with T1DM who have a well-controlled glycaemic profile manifest similar placental pathologies to those with sub-optimal glycaemic control [258]. Glucose-controlling agents such as metformin have demonstrated to impact miRNA expression in EVs and various *in vitro* and *in vivo* models [259–263]. With T2DM being associated with maternal adiposity and thus increased influence of lipids and adipokines [118], this further exemplifies the differences in the diabetic milieu across the various diabetic subtypes. Indeed, other maternal macro- and micronutrients that are altered in maternal diabetes, such as folate and vitamin B12 levels, have capacity to alter placental development and miRNA expression [264–266]. In addition, maternal ethnicity, diet, exercise, medication, infection, age, socioeconomic status and gestational age have all been identified as factors which influence placental miRNA regulation [267].

It is difficult to elucidate whether changes to the placental miRNA profile are a cause or a consequence of maternal diabetes in human clinical studies. *In vitro* findings show that mild hyperglycaemia directly alters the release of EV-miRNAs, which may explain why miRNAs are altered in the placenta and maternal circulation of pregnancies complicated by diabetes [268]. However, *in vivo* findings show that continued infusion of EVs from GDM pregnancies causes mice to develop glucose intolerance and altered tissue miRNA profile, compared with EVs from healthy pregnancies [269]. As such, these findings suggest that changes in miRNA regulation may be both a cause and a consequence of maternal diabetes.

Most studies to date have investigated the effects of GDM on placental miRNA profiling but not T1DM or T2DM. Given the difference in the underlying pathophysiology and clinical demographics of people with GDM, T1DM and T2DM, further research is required to profile placental miRNAs in all types of maternal diabetes [267].

### Circulating microRNAs

To our knowledge there are currently no published studies reporting miRNA profiles in the circulation of pregnant individuals with PGDM, however several EV-encompassed miRNAs have been shown to be altered in the maternal circulation in pregnancies complicated by GDM [200,270]. Whilst the tissue source of the majority of miRNAs in maternal circulation in GDM is unknown, it is likely that they originate from both maternal and placental tissue.

Indeed, this is supported by the observation that whilst individuals with GDM have lower circulating levels of placenta-derived small EVs compared with pregnant individuals without diabetes, overall, the level of EVs in maternal circulation is higher in GDM compared with those without diabetes [271]. This could suggest that the diabetic environment may be altering the release of various maternal organ EVs and their miRNA cargo into the circulation. Indeed, it has been shown that the hyperglycaemic component of the diabetic environment can modulate EV secretion and their miRNA cargo [195]. Some studies suggest that these changes in EV-miRNA cargo are protective adaptations against a hyperglycaemic environment [270]. However, it has also been postulated that EV-miRNAs may be involved in the pathogenesis of GDM where changes to EV-miRNA profiles have been detected in early pregnancy, prior to the diagnosis of GDM [200]. It has also been reported that maternal circulating miRNA expression in GDM pregnancies may differ depending on sex [272]. It remains to be established whether miRNAs that circulate to the placenta contribute towards altered placental development in pregnancies complicated by maternal diabetes, and if so, whether they originate from specific maternal organs. Considering the tissue-of-origin of circulating miRNAs may help to delineate this.

Moreover, the proportional reduction in placenta-derived small EVs in the maternal circulation of individuals with GDM could also suggest that the diabetic environment reduces placental tissue EV release and biogenesis. Studies have shown that placenta-EV miRNA profile is altered in pregnant people with maternal diabetes. The functional effects of these miRNAs have been associated with cell proliferation, migration, angiogenesis, inflammation and glucose metabolism (Table 2) [199,200,273–276]. It remains to be established whether the effects of these placenta-derived EV-miRNAs in pregnancies complicated by maternal diabetes are limited to maternal organs or if they extend to fetal tissues. However, with placenta-derived EVs being able to interact with various maternal metabolic tissues and regulate maternal glucose homeostasis [194–196], it is possible that the altered placental EV release and miRNA content stimulated by the diabetic milieu may lead to changes in maternal metabolism and contribute towards GDM. Recent evidence shows that placenta-derived EVs from GDM pregnancies manifest an altered miRNA profile which is associated with aberrant insulin signalling and altered primary skeletal muscle cell insulin-stimulated migration and glucose uptake [199,200,276] (Table 2). As such, these findings suggest that placenta-derived EV-miRNAs likely play a role in influencing maternal metabolism in GDM.

Not only is the secretion and miRNA cargo of placenta-EVs affected by maternal diabetes, but evidence also suggests that EV production by maternal metabolic organs may be affected, which can in turn influence placental metabolism and maternal health in GDM [189]. Indeed, Nair et al. have demonstrated that continuous infusion of human maternal EVs from GDM pregnancies into healthy non-pregnant mice reduces pancreatic islet glucose-stimulated insulin secretion and promotes glucose intolerance, where skeletal muscle miRNA expression and glucose sensitivity are altered [277]. EVs derived from the adipose tissue of pregnant individuals with GDM have demonstrated to impact glucose metabolism in placental cells, causing alterations in glycolytic, gluconeogenic and glycogen storage processes [189]. While this study does not ascribe the effects of these adipose tissue-EVs to miRNA activity, glucose-sensitive miRNAs have been reported in adipose tissue which may impact insulin sensitivity [278]. In people with GDM, adipose tissue miRNA profile is altered; miR-222 was found to be up-regulated and suggested to be a key regulator of insulin resistance [242]. Placenta-derived EVs from individuals with GDM also showed altered expression levels of this miRNA [199]. Another recent study suggested that maternal visceral fat thickness may predict the risk of developing GDM via adipose tissue derived EV-miR-148 family signalling [279]. Interestingly, it has been previously reported that miR-148 is altered in placental tissue of those with GDM and T2DM, with its targets associated with insulin signalling and glucose metabolism [209]. As such, maternal diabetes has a significant influence on maternal organ EV-miRNA cargo which could contribute towards altered feto-placental development.

## microRNAs and altered fetal growth in pregnancies complicated by maternal diabetes

The miRNAome at the maternal–fetal interface has a direct influence on fetal growth and development [280–282]. Maternal diabetes impacts the maternal-fetal miRNAome by influencing placental miRNA expression and EV-mediated interorgan communication, which in turn has been linked to altered fetal growth [188,277]. Although lacking, a few studies have determined associations between fetal growth and miRNA expression levels in the maternal circulation and in placental tissue in pregnancies complicated by maternal diabetes (Table 3). The epidermal growth factor receptor (EGFR) pathway has been identified as a functional target of various miRNAs which are altered in the placenta of GDM pregnancies resulting in fetal overgrowth [283]. It is known that this signalling pathway plays a key role in placental and fetal development. As such, it is possible that the miRNAs identified to be associated with altered fetal growth in pregnancies complicated by maternal diabetes are key for regulating optimal fetal development (Table 3).

## microRNAs and altered fetal development and offspring health in pregnancies complicated by maternal diabetes

Maternal diabetes is associated with adverse offspring health outcomes, including an increased risk of developing cardiometabolic complications throughout life compared with offspring from uncomplicated pregnancies [25–27]. Increasing evidence suggests that maternal circulating factors may play a role in the development of these adverse adaptations. For example, it has been shown that injection of maternal EVs from diabetic mice into healthy pregnant mice contributes towards fetal cardiac developmental deficiency [287]. Similar findings have been demonstrated with fluorescently-labelled maternal EVs in a diabetic mouse model, where the maternal EVs were able to cross the placenta and increase the risk of congenital heart defects in the offspring [288]. Although not specific to maternal diabetes, a recent investigation found that visceral adipose tissue EVs from obese mice contributed towards reduced fetal cardiac function in healthy lean pregnant mice by altering events in the placenta [289]. Although these studies do not investigate the role of EV-miRNA cargo contributing towards these adverse effects, increasing evidence supports the role of miRNAs in epigenetic programming and cardiovascular disease [290]. Indeed, studies have shown that offspring of pregnancies affected by maternal diabetes demonstrate altered fatty acid oxidation and glucose metabolism as a result of altered miRNA regulation [291,292]. Specifically, a recent pilot study has characterised a set of miRNAs associated with diabetes and cardiovascular disease to be dysregulated in the circulation of children exposed to GDM *in utero* [293]. Interestingly, many of these miRNAs have demonstrated to be altered in the maternal circulation and placental tissue of GDM pregnancies discussed in this review. Other animal studies have shown that baboon offspring exposed to GDM *in utero* also demonstrate altered cardiac miRNA expression, thereby increasing the risk of cardiac hypertrophy, myocardial infarction and cardiomyopathy [294]. Another study showed that fetal cardiac tissue from pregnant diabetic-induced mouse models demonstrated let-7e-5p, miR-139-5p, and miR-195-5p up-regulation and increased cardiac wall thickness [295].

Suboptimal maternal nutrition can impact offspring susceptibility to metabolic disease and cardiovascular dysfunction in a sex-specific manner [296–300]. Indeed, it has been shown that suboptimal maternal nutrition during pregnancy can programme offspring lipid metabolism and insulin resistance, thus contributing towards the development of T2DM [301]. GDM has been demonstrated to alter fetal lipid metabolism in a sex-dependent manner via miRNA activity, whereby the liver of male rat fetuses demonstrated down-regulated miR-130 expression, resulting in PPARγ up-regulation. Conversely, female rat fetal liver exclusively demonstrated miR-9 down-regulation and PPARδ up-regulation in response to GDM [302]. The sex-specific influence of GDM on fetal hepatocyte miRNA regulation is becoming increasingly apparent, therefore our understanding of the contribution of miRNAs to the development of metabolic disorders, adipogenesis, obesity and fatty liver disease onset in offspring of GDM pregnancies is continuously progressing [303]. However, further research is needed to elucidate whether the sex-dependent effects on miRNA expression in male and female offspring of pregnancies complicated by maternal diabetes are due to increased vulnerability or advanced environmental adaptation to hyperglycaemia [251].

Evidence also suggests that miRNAs modulate fetal cerebrovascular development. It is theorised that dysregulation of miRNAs contributes to the increased risk of neurodevelopmental disorders in diabetic pregnancies [293,304,305]. To date, most studies investigating the effects of maternal diabetes on offsprings’ long-term health have only focused on GDM. In recent years, it has become apparent that postpartum circulating EV-miRNA profile is altered in lactating individuals with T1DM, where dysregulated miRNAs have been associated with disease progression and inflammation, thus increasing offspring risk of immune-mediated diseases [306]. However, the long-term impact of maternal PGDM on offspring health and development requires further research.

## microRNAs and maternal cardiometabolic health in pregnancies complicated by maternal diabetes

GDM is known to increase the risk of adverse maternal outcomes, both during pregnancy and postpartum [307]. Preeclampsia is a frequent complication of pregnancies complicated by maternal diabetes; a condition known as high blood pressure and proteinuria during the third trimester of pregnancy which can lead to reduced placental blood flow, resulting in a lack of nutrient and oxygen exchange at the feto-placental interface. As well as affecting the developing fetus, preeclampsia is associated with increased maternal risk of cardiovascular and cerebrovascular disease onset postpartum [308,309]. miRNAs may play a role in the pathophysiology of preeclampsia. Indeed, a dysregulated miRNA profile has been identified in preeclamptic placentae, where miR-106a∼363 cluster expression is altered compared with healthy uncomplicated pregnancy [310]. C19MC miRNAs are also implicated in preeclampsia, where placental miR-516-5p, miR-517*, miR-520a*, miR-525 and miR-526 expression are up-regulated [311]. More recently, there is a call to further identify a robust miRNA biomarker profile that is uniquely altered in people with preeclampsia and determine whether their expression is resolved post-recovery [312].

Individuals with GDM are also at higher risk of developing T2DM postpartum [6]. It has been demonstrated that postpartum levels of several circulating miRNAs, including miR-16-5p, miR-17-5p, miR-29a-3p, miR-195-5p and miR-369-3p, are associated with postpartum diabetes onset in people with GDM [313,314]. In a 15-year follow-up study, circulating miR-24-3p expression, along with maternal weight and BMI, has also been associated with the future progression of dysglycaemia in individuals with GDM postpartum [315]. Interestingly, a mediterranean diet has demonstrated to improve insulin sensitivity and inflammation in people with GDM post-partum through the regulation of miR-222 and miR-103 [316]. Whilst the mechanism and relationship between miRNAs and postpartum diabetes status remains to be established, the altered levels of miRNAs in circulation could potentially contribute towards reduced insulin sensitivity by influencing maternal organs, for example down-regulation of miR-369 has previously been reported in the pancreas in T2DM [317]. miR-24 expression levels have also been shown to both inversely correlate with Hba1C levels and to influence endothelial cell function in T2DM [318]. This corroborates with the observation that people with GDM have increased postpartum endothelial dysfunction [319], and various endothelial cell models demonstrating miRNA dysregulation under GDM conditions.

In addition to increased rates of T2DM following a GDM pregnancy, the risk of future cardiovascular diseases is increased by 2-fold for individuals diagnosed with GDM compared with those who experience healthy, uncomplicated pregnancies. This includes risk of stroke, ischemic heart disease and heart failure [320]. The mechanisms linking GDM to post-partum cardiac health remain to be established but 11 maternal circulating miRNAs (miR-13p, miR-20a-5p, miR-20b-5p, miR-23a-3p, miR-100-5p, miR-125b-5p, miR-126-3p, miR-181a-5p, miR-195-5p, miR-499a-5p and miR-574-3p) associated with cardiovascular disease have been found to be increased in the first trimester of GDM pregnancies compared with healthy uncomplicated pregnancy [321]. These findings therefore suggest that miRNAs may play a role in postpartum adverse maternal cardiometabolic health observed in GDM pregnancies; however, further studies are needed to establish the aetiology behind these associations.

## microRNAs in the diagnosis and management of pregnancies complicated by maternal diabetes

miRNAs have a clear role in the progression of maternal and fetal complications in pregnancies affected by maternal diabetes. Whilst further research is still needed to elucidate the pleiotropic effects and mechanisms or miRNAs, their biomarker potential to detect adverse maternal and fetal outcomes in pregnancies complicated by maternal diabetes is well-recognised. Not only do miRNAs increase understanding of underlying pathophysiology and altered target genes associated with diseases, but they may also provide an alternative means of non-invasive testing. GDM is usually diagnosed through an oral glucose tolerance test (OGTT) at weeks 24–28 gestation when pregnancy is at an advanced stage, meaning minimal interventions can be implemented to avoid or manage adverse maternal and fetal outcomes [72]. However, recent studies are suggesting that earlier detection and treatment is associated with better maternal and neonatal outcomes [322]. There is currently a lack of consensus on the gold standard diagnostic criteria that should be used for GDM, which has led to heterogenous guidelines and confusion about the prevalence of GDM worldwide [323]. As such, many people with hyperglycaemia go undetected throughout pregnancy. There are certain maternal circulating miRNAs that have been identified as potential diagnostic biomarkers for GDM; however, intra-study reproducibility is an essential factor to consider when identifying miRNAs as biomarkers [72,324,325]. There are currently only six miRNAs (miR-195, miR-330, miR-342, miR-520h, miR-657, miR-1323) that have been similarly differentially expressed in people with GDM across multiple studies, with these studies using a range of methodologies and involving individuals of different populations and age range [267]. Most of these miRNAs have been identified to be altered in the placenta of people with GDM (Table 1). Using miRNAs as biomarkers for GDM diagnosis would provide another layer of screening for improved consensus [267]. This would allow for adverse maternal and fetal outcomes to be detected at an earlier stage of pregnancy and thus allow more time for intervention strategies to be implemented, such as exercise, diet and medication [72]. It has been shown that exercise during pregnancy can alter the plasma miRNA profile in individuals with GDM, giving rise to potential biomarkers that may be used to detect GDM risk [326]. Evidence also suggests that maternal exercise during pregnancy may improve female offspring hepatic metabolism by modulating miRNA activity and reverse the dysregulated fetal cardiac miRNA profile identified in GDM pregnancies [327,328].

Since maternal diabetes increases the risk of neonates being classed as LGA and predisposes them to cardiometabolic complications throughout life, infants of pregnancies complicated by maternal diabetes would benefit from primary prevention strategies. Identifying pregnancies at risk LGA would also allow suitable birth planning and improved clinical management of mother and offspring throughout pregnancy. Additionally, follow-up screening of the neonatal miRNA profile may provide better identification of congenital abnormalities that are associated with pregnancies complicated by maternal diabetes, such as cardiovascular and cerebrovascular complications [293]. Maternal diabetes is also associated with an increased risk of postpartum maternal cardiometabolic disease and cancer. Thus, identifying the altered maternal miRNA profiles associated with these complications would also improve clinical management of maternal health after pregnancy.

Currently, there is a lack of study reproducibility of miRNAs being used as biomarkers. Sample collection, storage, isolation and processing methodologies all influence miRNA quality and stability [329–331]. Therefore, improving consensus on the appropriate methods to use for miRNA biomarker studies would increase reproducibility.

## Conclusion

miRNAs play a key role in regulating optimal placental and fetal development during pregnancy, with their dysregulation contributing towards altered placental growth and metabolism in pregnancies complicated by maternal diabetes. To date, the vast majority of studies have focused on the effect of GDM on the placental miRNA profile; however with their unique underlying placental pathophysiology, future studies should further investigate the effects of T1DM and T2DM on placental miRNA expression. Moreover, not only do miRNAs have a direct effect on placental regulation and fetal development during pregnancy, but they may also serve as potential biomarkers for health disorders in offspring. Increasing evidence also suggests circulating miRNAs may predict maternal risk of developing GDM and adverse outcomes postpartum. However, further research is needed to improve the reproducibility of miRNAs as biomarkers in pregnancies complicated by maternal diabetes. With maternal diabetes being associated with suboptimal placental development, altered fetal growth and adverse health outcomes for mother and offspring, miRNAs provide a non-invasive screening alternative into disease pathology and improved clinical management of maternal and fetal health during pregnancy.

## Data Availability

No new data were generated or analysed in support of this research.

## Open Access

Open access for this article was enabled by the participation of University of Leeds in an all-inclusive *Read & Publish* agreement with Portland Press and the Biochemical Society under a transformative agreement with JISC.

## Abbreviations

BM: basal plasma membrane
C19MC: chromosome 19 miRNA cluster
CGM: continuous glucose monitoring
EOT2D: early onset T2DM
EV: extracellular vesicle
FATP: fatty acid transport protein
GLP-1: glucagon-like peptide-1
OGTT: oral glucose tolerance test
SNAT: sodium-coupled neutral amino acid transporter
T1DM: Type 1 diabetes mellitus
T2DM: Type 2 diabetes mellitus
